# A screen for inhibitory peptides of hepatitis C virus identifies a novel entry inhibitor targeting E1 and E2

**DOI:** 10.1038/s41598-017-04274-8

**Published:** 2017-06-21

**Authors:** Peiqi Yin, Ling Zhang, Fei Ye, Yao Deng, Sha Lu, Yi-Ping Li, Leiliang Zhang, Wenjie Tan

**Affiliations:** 1 MOH Key Laboratory of Systems Biology of Pathogens, Institute of Pathogen Biology, Chinese Academy of Medical Sciences and Peking Union Medical College, Beijing, 100730 China; 2 Key Laboratory of Medical Virology, Ministry of Health, National Institute for Viral Disease Control and Prevention, China CDC, Beijing, 102206 China; 3 Department of Medical Microbiology, Inner Mongolia Medical University, Hohhot, 010059 China; 4 Institute of Human Virology and Key Laboratory of Tropical Disease Control of Ministry of Education, Zhongshan School of Medicine, Sun Yat-sen University, Guangzhou, 510080 China

## Abstract

Hepatitis C virus (HCV) entry into hepatocytes is a multistep process that represents a promising target for antiviral intervention. The viral envelope protein E1E2 plays a critical role in HCV entry. In this study, we sought to identify peptide inhibitors of HCV by screening a library of overlapping peptides covering E1E2. Screening the peptide library identified several novel anti-HCV peptides. Four peptides from glycoprotein E2 were selected for further investigation. The 50% effective dose (ED50) was approximately 5 nM for each peptide. Our data indicated that these peptides inhibited HCV entry at the post-attachment step. Moreover, these peptides blocked cell-to-cell transmission of HCVcc and had broad-spectrum antiviral effects on HCVcc. These peptides exhibited combination inhibitory effects on HCVcc infection when combined with IFN-α2b or anti-CD81 antibody. Interestingly, we observed that E2-42 associated with E1 and E2. Our results indicate that E2-42 inhibits HCV entry via E1 and E2. These findings suggest a new avenue for HCV therapeutic development.

## Introduction

Chronic hepatitis C virus (HCV) infection is a major cause of chronic liver diseases, including chronic hepatitis, liver cirrhosis and hepatocellular carcinoma (HCC). Recent estimates suggest that 64–103 million people are infected with HCV worldwide^1^. No effective vaccine against HCV infection is available, and treatment with PEGylated interferon alpha (PEGIFN-α) plus ribavirin is associated with a response rate of only approximately in patients infected with the most prevalent genotype, genotype 1. Although recently developed direct-acting antivirals (DAAs), including inhibitors of HCV NS3/4 A protease (Boceprevir, Telaprevir, and Simeprevir), NS5A (Daclatasvir, Ledipasvir, and Ombitasvir), and NS5B polymerase (Sofosbuvir, Mericitabine, and Dasabuvir), have revolutionized hepatitis C treatment, several important challenges remain^2, 3^. Potential adverse effects, the risks of selecting drug-resistant mutants, drug-drug interactions, and difficult-to-treat populations are important issues that limit the availability of DAAs to all HCV-infected patients^3^. More importantly, the high cost of DAAs restricts their accessibility in most parts of the world. Thus, continuous development of alternative potential inhibitors that target different steps of the HCV life cycle, including viral entry, is urgently needed.

As a member of the Flaviviridae family, HCV is an enveloped positive-sense single-strand RNA virus. The viral genome encodes structural (core, E1, E2 and p7) and non-structural (NS2, NS3, NS4A, NS4B, NS5A and NS5B) proteins^4^. Of these, E1 and E2 are important for HCV entry. HCV entry is a multi-step process that begins with the attachment of a viral particle to the cell surface via attachment factors, followed by a complex process involving a series of cellular entry co-receptors, including scavenger receptor class B type I (SR-BI)^5^, CD81^6^, claudin-1^7^, occludin^8^, and the receptor tyrosine kinases epidermal growth factor receptor (EGFR)^9^, ephrin receptor A2^9^, Niemann-Pick C1-like 1 (NPCL1)^10^, and transferrin receptor 1 (TfR1)^11–14^. The viral particle enters the host cell via clathrin-mediated endocytosis, which is followed by viral genome release, translation, replication, assembly, and exit of progeny virions to complete the HCV life cycle^14^.

Due to its multi-step nature, HCV entry is an attractive target for inhibiting HCV. Monoclonal antibodies and small molecules inhibiting HCV entry have been considered^12, 13^. Short peptides derived from viral envelope sequences that contain membrane-transiting motifs have been designed to inhibit virus entry into cells. For example, a peptide derived from the amino acids 710 to 725 of the HCV E2, inhibits HCV pseudoparticle infection^15^. The amphipathic α-helical peptide C5A derived from the membrane anchor domain of the HCV NS5A protein exhibits significant inhibitory effects against HCV infection *in vitro* ^16^. Because the strategy of developing antiviral peptides based on viral membrane proteins is feasible, systematic screening of anti-HCV peptides from HCV E1E2 is promising.

In this study, we screened a peptide library from JFH1 E1E2 and identified four novel HCV inhibitory peptides functioning at the post-attachment and viral spread steps. We further determined that one peptide, named E2-42, interacted with E1 and E2. Overall, our work opens a new avenue for screening anti-HCV peptides and uncovers potential anti-HCV agents.

## Results

### Identification of antiviral peptides from HCV E1E2

**Figure 1:**
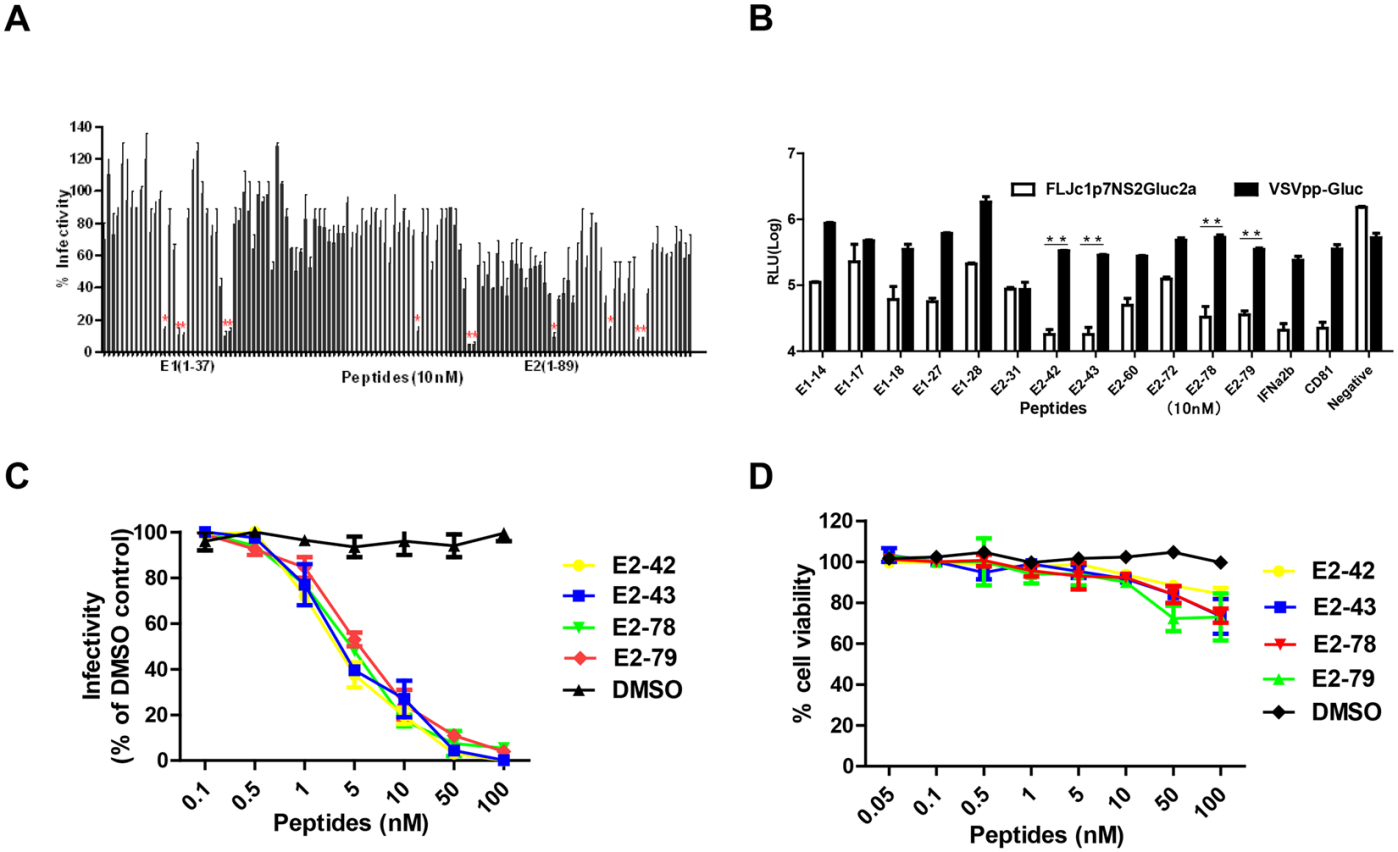
Identification of HCV E1E2-derived peptides with antiviral activity against HCVcc infection. (**A**) Effects of E1E2-derived peptides on HCV E1E2-mediated infection. HCVcc Jc1P7NS2Gluc2a was used to screen for antiviral activity. HCVcc was incubated with 10 nM peptides for 1 h prior to addition to Huh7.5/CD81 cells, and Gaussia luciferase activity was assayed 72 h later. The results were normalized to DMSO-treated cells. (**B**) Characterization of the specificity of inhibitory peptides against HCVcc infection. HCVcc and VSV-Gpp (VSV-G-pseudotyped lentivirus) were incubated with 10 nM peptides for 1 h. Then, Huh7.5/CD81 cells were inoculated for 4 h with virus/peptide mixtures, and Gaussia luciferase activity was assayed 72 h later. IFN-α and anti-CD81 antibodies were used as positive controls, and DMSO was used as a negative control. (**C**) The ED50 values of peptides were calculated based on their inhibition of HCVcc activity at different peptide concentrations. (**D**) Cytotoxicity of peptides against Huh7.5/CD81 cells by MTT assay. Each bar represents the average of triplicate data points, with the standard deviation indicated by the error bars.

To screen peptides that inhibit HCV infection, a peptide library of 126 overlapping peptides (15-mers offset by 10 amino acids for E1, by 11 amino acids for E2) covering the envelope protein E1E2 of the HCV JFH1 strain (GenBank no. AB047639) was designed. The peptides were incubated with Jc1P7NS2Gluc2a for 1 h, and then the virus was used to infect Huh7.5-CD81 cells. In the preliminary trials of antiviral peptide screening, 12 peptides inhibited HCVcc infection by more than 80% at 10 nM (Fig. 1A). To confirm that these 12 peptides specifically inhibited HCV infection, VSVpp-Gluc was used as a control virus. As shown in Fig. 1B, four peptides from E2 (E2-42, E2-43, E2-78, E2-79) significantly decreased HCV infection compared to VSVpp-Gluc; IFN-α2b and CD81 antibodies were used as the positive control (P < 0.01). Four inhibitory peptides from E1 (E1-17, E1-18, E1-27, and E2-28) were reported in the AVPdb database^17^ (Table 1). Thus, we focused on the other four novel peptides (E2-42, E2-43, E2-78, and E2-79).

**Table 1:** Anti-HCV peptides in our screen.

Peptides	Sequence (15 aa)	Position (aa-aa)	AVPid	Sequence
E1-14	GLRTHIDMVVMSATF	257–271		
E1-17	CSALYVGDLCGGVML	272–286	AVP1461	GSATLCSALYVGDLCGSV
E1-18	VGDLCGGVMLAAQVF	277–291	AVP1462	ALYVGDLCGSVFLVGQLF
E1-27	MMMNWSPTATMILAY	322–336	AVP1469	MMNWSPTAALVVAQLLRI
E1-28	SPTATMILAYVMRVP	327–341	AVP1469	MMNWSPTAALVVAQLLRI
E2-31	VCGPVYCFTPSPVVV	504–518		
E2-42	QGSWFGCTWMNSTGF	548–562		
E2-43	FGCTWMNSTGFTKTC	552–566		
E2-60	WHYPCTVNFTIFKIR	620–634		
E2-72	PLLHSTTEWAILPCT	668–682		
E2-78	GLLHLHQNIVDVQYM	692–706		
E2-79	LHQNIVDVQYMYGLS	696–710		

Next, we investigated whether E2-42, E2-43, E2-78, and E2-79 inhibit HCV infection in a dose-dependent manner. As shown in Fig. 1C, the four peptides blocked HCV infection with a 50% inhibitory concentration (IC50) of 1–5 nM. Then, we evaluated the cytotoxicity of the four peptides. The four polypeptides had no effect on the viability of Huh7.5-CD81 cells at concentrations of 10 nM, indicating that the anti-HCV effects of E2-42, E2-43, E2-78, E2-79 were not due to toxicity.

### E2-42, E2-43, E2-78 and E2-79 have broad-spectrum inhibitory effects on different HCV genotypes

**Figure 2:**
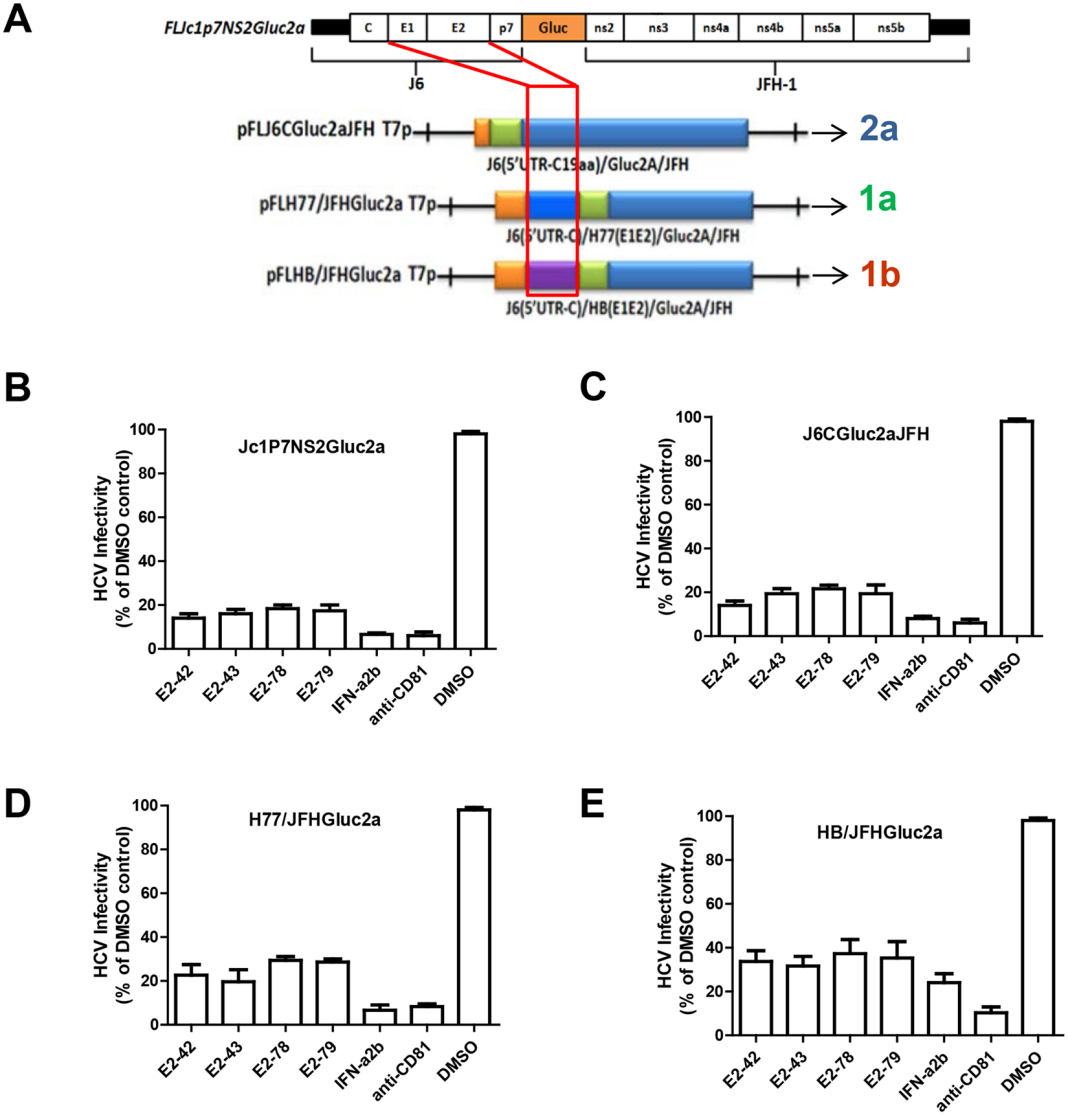
Broad-spectrum inhibitory effects of the peptides against different chimeric HCVcc clones. **(A**) Schematic diagram of the different chimeric HCV infectious clones. (**B**) Huh7.5/CD81 cells were treated with 10 nM peptides and different chimeric HCVcc clones for 1 h. Then, the cells were incubated for another 72 h, and Gaussia luciferase activity was measured. The antiviral effects of the peptides on chimeric HCVcc J6CGluc2aJFH (genotype 2a) (**B**), J6CGluc2a (**C**), H77/JFHGluc2a (genotype 1a/2a) (**D**), and HB/JFHGluc2a (genotype1b/2a) (**E**) were determined. Each bar represents the average of triplicate data points, with the standard deviation represented by the error bar.

**Figure 3:**
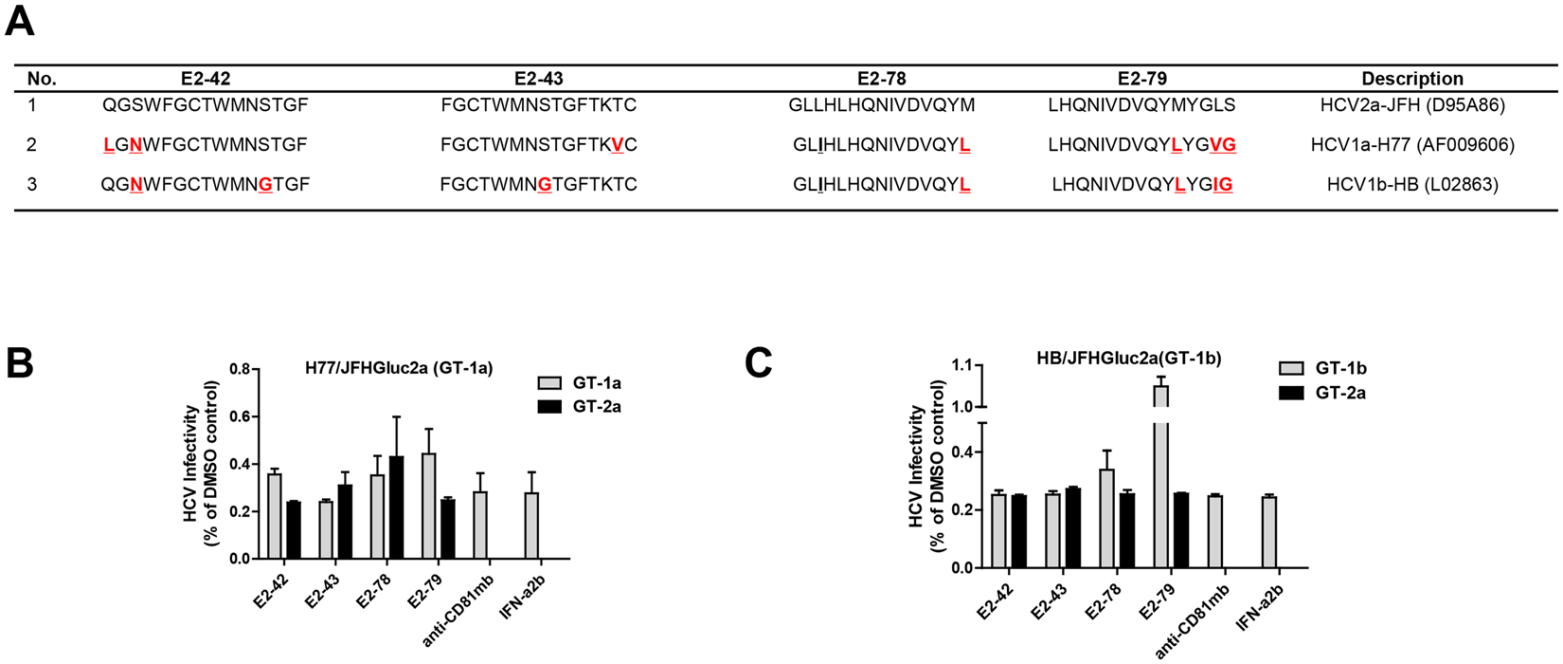
The inhibitory effects of the peptides on different genotypes of HCVcc. (**A**) Amino acid sequences of peptides from various HCV genotypes. (**B**,**C**) Inhibitory effects of peptides from genotypes 1a and 2a against chimeric HCVcc H77/JFHGluc2a (**B**) and HB/JFHGluc2a (**C**). Each bar represents the average of triplicate data points, with the standard deviation indicated by the error bar.

To determine the antiviral activity of E2-42, E2-43, E2-78, and E2-79 against different HCV genotypes and subtypes, the four peptides (10 nM) were incubated with different genotypes of HCVcc, including genotype 2a, Jc1p7NS2Gluc2a and J6CGluc2a; genotype 1a/2a, H77/JFHGluc2a; and genotype 1b/2a, HB/JFHGluc2a (Fig. 2A). The virus/peptide mixtures were then used to infect Huh7.5-CD81 cells. The four peptides from E2 exerted a broad spectrum of antiviral effects against HCV but different inhibitory efficiencies for different genotypes of HCVcc. Peptides E2-42 and E2-43 had slightly higher efficiency than peptides E2-78 and E2-79. For genotypes 2a, 1a, and 1b, the inhibition rates were 80%, 75% to 80%, and 60% to 70%, respectively (Fig. 2B,C,D and E). The genotype-dependent inhibitory effects may due to differences in amino acid composition among genotypes (Fig. 3A). Next, we synthesized conservative genotype 1a and 1b peptides and evaluated the inhibition ratio for the corresponding genotypes of HCVcc. As shown in Fig. 3B and C, the inhibitory effect of E2-79 from 1a or 1b was significantly decreased compared to that from genotype 2a, indicating that the differences in amino acid composition between genotypes in E2-79 may play an important role in its antiviral effect.

### The four peptides inhibit HCV entry and cell-to-cell transmission

**Figure 4:**
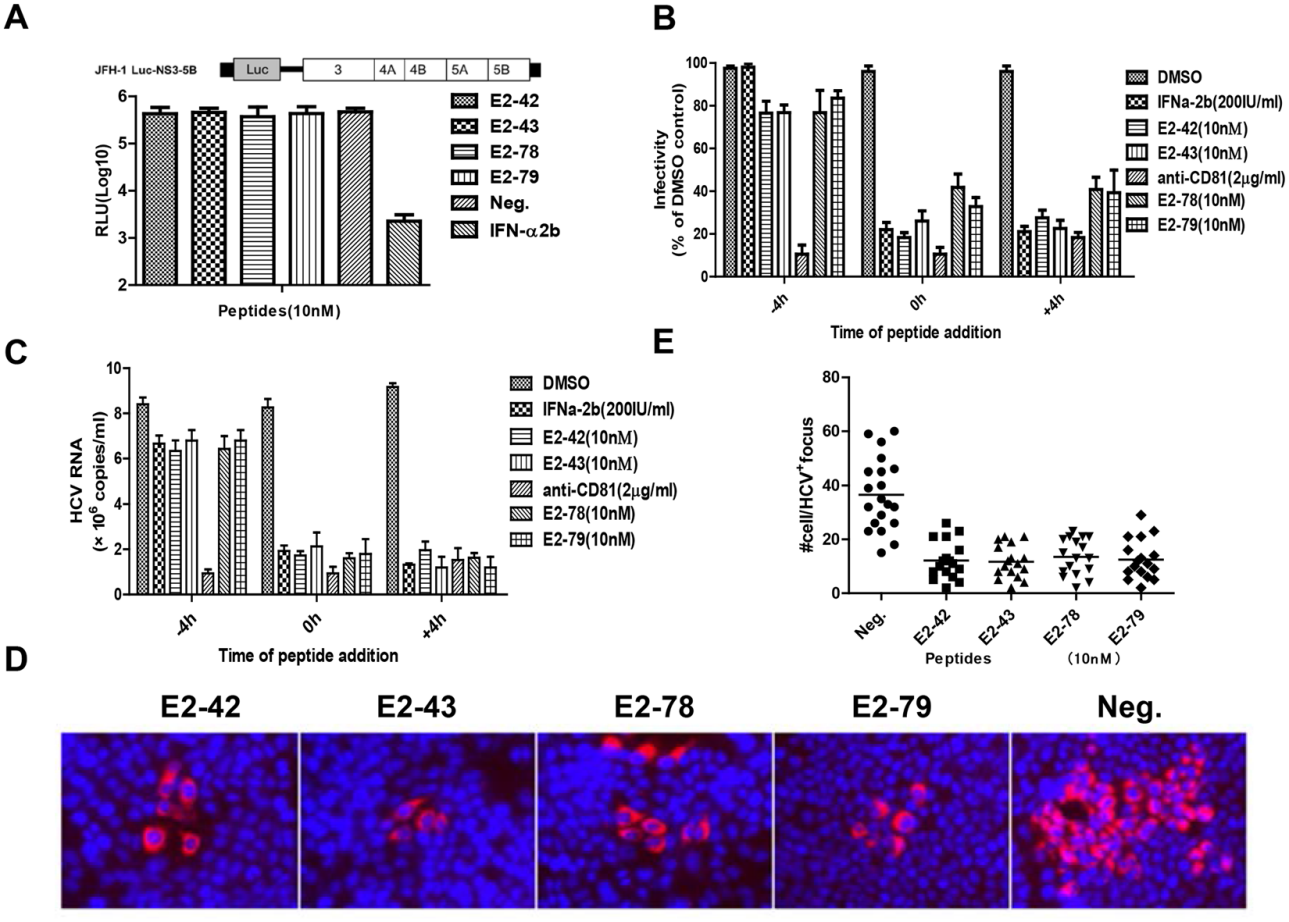
The peptides selectively inhibit cell entry in a virus post-attachment step but do not affect viral RNA replication. (**A**) The inhibitory effect of the peptides on HCV replication in JFH1Luc-NS3-5B replicon cell lines. JFH1Luc-NS3-5B replicon cells were incubated with 10 nM peptides for 2 h, and Gaussia luciferase activity was assayed 72 h later. DMSO-treated cells were used as a negative control. (**B**,**C**) Peptides were added 4 h before, with or after infection with HCVcc. Peptide or anti-CD81 antibody was added to the cells for 4 h and then was removed before inoculation (−4 h), together with virus at the time of inoculation (0 h), or 4 h after virus had been removed by washing (+4 h). The cells for −4 h and 0 h were washed after 4 h incubation and replaced with virus/peptide-free medium, whereas for + 4 h condition the peptides or anti-CD81 antibody remained in the cell cultures throughout the experiment. RNA replication was quantified by Gaussia luciferase assays 72 h after infection (**B**), and HCV RNA copies were assayed by qRT-PCR (**C**). The luciferase results for DMSO, IFN-α2b and anti-CD81 antibody-treated cells served as controls. (**D**,**E**) Characterization of the effects of the peptides on HCVcc transmission. Huh7.5/CD81 cells were seeded at 5 × 10^4^/well in 24-well plates the day before infection. The cells were infected with 0.1 FFU/cell mixed with peptides for 2 h, and then the cells were covered with DMEM containing low melting-agarose for culture. After 72 h, the cells were fixed for an immunofluorescence assay. HCV-infected cells were detected by immunofluorescence microscopy (**D**), and the number of HCV-positive cells per focus was enumerated in 20 foci (**E**). The results of DMSO treatment served as a control. Each bar represents the average of triplicate data points, with the standard deviation represented by the error bar.

To evaluate the effects of peptides on HCV replication, we used an HCV replicon cell line called JFH1 Luc-NS3-5B. After treatment with peptides for 72 h, the replicon cells were lysed to measure luciferase activity. As shown in Fig. 4A, the peptides did not inhibit HCV RNA replication. To analyze whether the peptides had any effect on HCV entry, the peptides were incubated with Huh7.5-CD81 cells at different time points of Jc1P7NS2Gluc infection. The results clearly indicated a decrease in HCVcc infection when the peptides were present during and after virus infection based on luciferase activity measurements (Fig. 4B) and HCV RNA levels (Fig. 4C). Furthermore, there was no effect if peptides were added to the cells as a pretreatment. These results suggest that the four peptides inhibit HCV entry at the post-attachment step.

HCV is also spread via cell-to-cell transmission. After infection with HCVcc, progeny viruses are transmitted from donor cells to acceptor cells, resulting in focal areas of infection transmission. To determine if E2-42, E2-43, E2-78, or E2-79 inhibit cell-to-cell transmission of HCV, Huh7.5-CD81 cells were infected with HCVcc and overlaid with media containing 50 nM peptides and 1% agarose. After 72 h of infection, the cells were stained with HCV core antibody. Focus sizes were measured by counting the number of cells per focus. As shown in Fig. 4D and E, cell-to-cell transmission was blocked by the four peptides. Taken together, these results demonstrate that the four peptides prevent HCV entry and cell-to-cell transmission.

### Combination of inhibitory peptides with IFN-α2b or CD81 antibody

**Figure 5:**
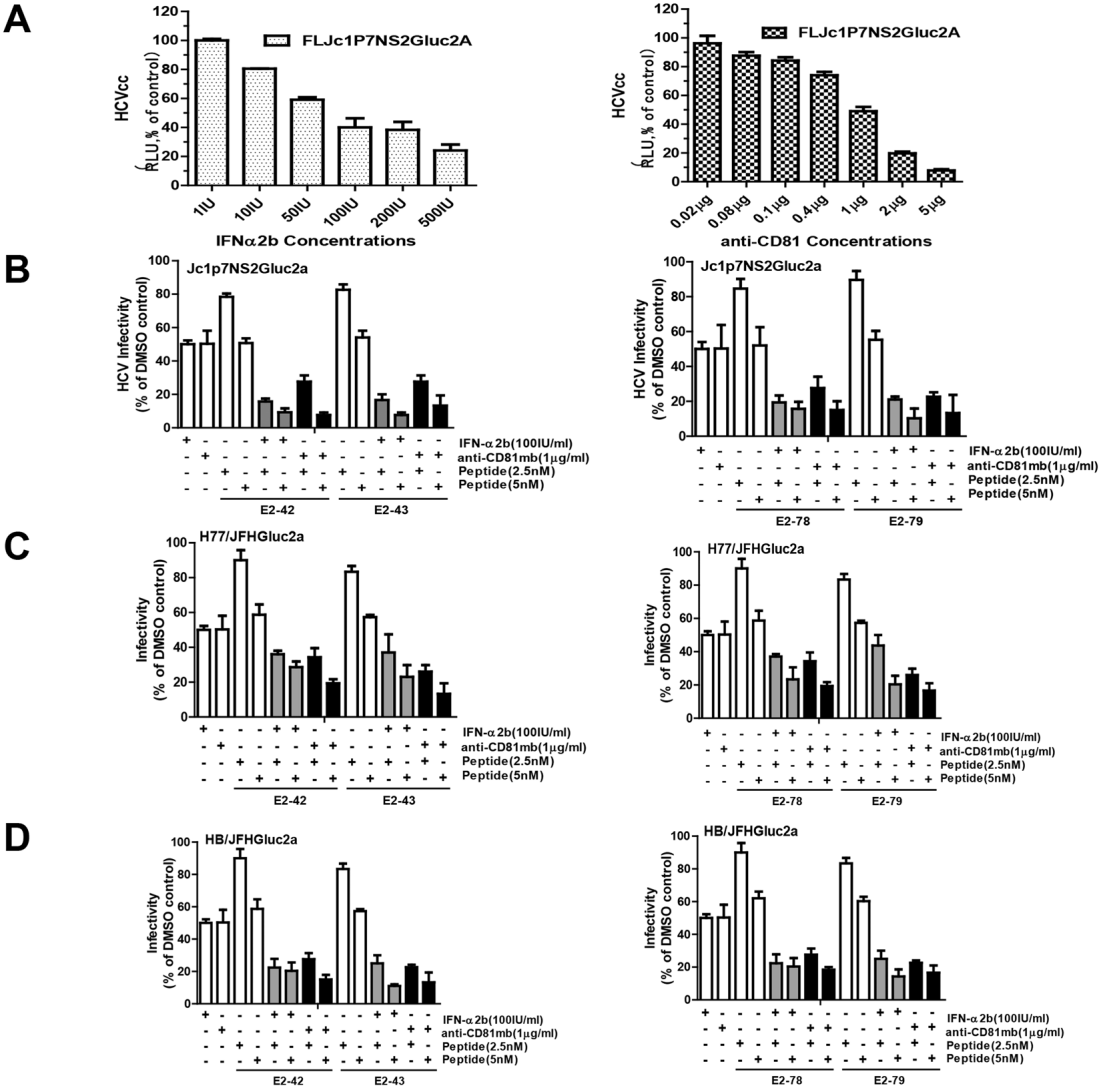
Analysis of the combination effects of the peptides combined with IFN-α2b and anti-CD81 antibodies. (**A**) Determination of the EC50 of IFN-α2b and anti-CD81 antibodies for HCVcc. Huh7.5/CD81 cells were seeded at 1 × 10^4^/well in 96-well plates the day before infection. The cells were infected with 0.05 FFU/cell mixed with serial dilutions of IFN-α2b and anti-CD81 antibodies. Gaussia luciferase activity was measured 72 h later. DMSO treatment served as a negative control. (**B**,**C**,**D**) The inhibitory effects of the peptides in combination with IFN-α2b or anti-CD81 antibody against HCV infection was assayed with chimeric HCVcc. Huh7.5/CD81 cells were treated with peptides, IFN-α2b or anti-CD81 antibodies and infected with different genotypes of chimeric HCVcc. The Gaussia luciferase activities of Jc1P7NS2Gluc2a (**B**), H77/JFHGluc2a (**C**), HB/JFHGluc2a (**D**) were assayed 72 h later. HCV infectivity was expressed as the percentage of the DMSO control. Each bar represents the average of triplicate data points, with the standard deviation represented by the error bar.

To determine if the inhibitory peptides from E2 complement other anti-HCV drugs, we investigated their antiviral activity in combination with IFN-α2b or CD81 antibody. First, we established that the IC50 of IFN-α2b was 100 IU/mL and that the IC50 of CD81 antibody was 1 μg/mL (Fig. 5A). Then, peptides at 2.5 nM or 5 nM in combination with IFN-α2b or CD81 antibody were incubated with genotype 2a HCVcc (Jc1p7NS2Gluc2a) for 2 h and added to Huh7.5-CD81 cells. After 4 h of infection, the virus mixture was removed, and luciferase was measured 72 h later. The results illustrated that the four peptides exhibited combination effects with antiviral IFN-α2b and CD81 antibodies for the inhibition of HCVcc (Fig. 5B). Similar results were observed for genotype 1a HCVcc (H77/JFHGluc2a) and genotype 1b HCVcc (HB/JFHGluc2a) (Fig. 5C and D). Taken together, these results indicate that the four peptides from E2 have potential for combination therapy with IFN-α2b and CD81 antibody.

### E2-42 binds HCV envelope proteins but does not interact with HCV receptors

**Figure 6:**
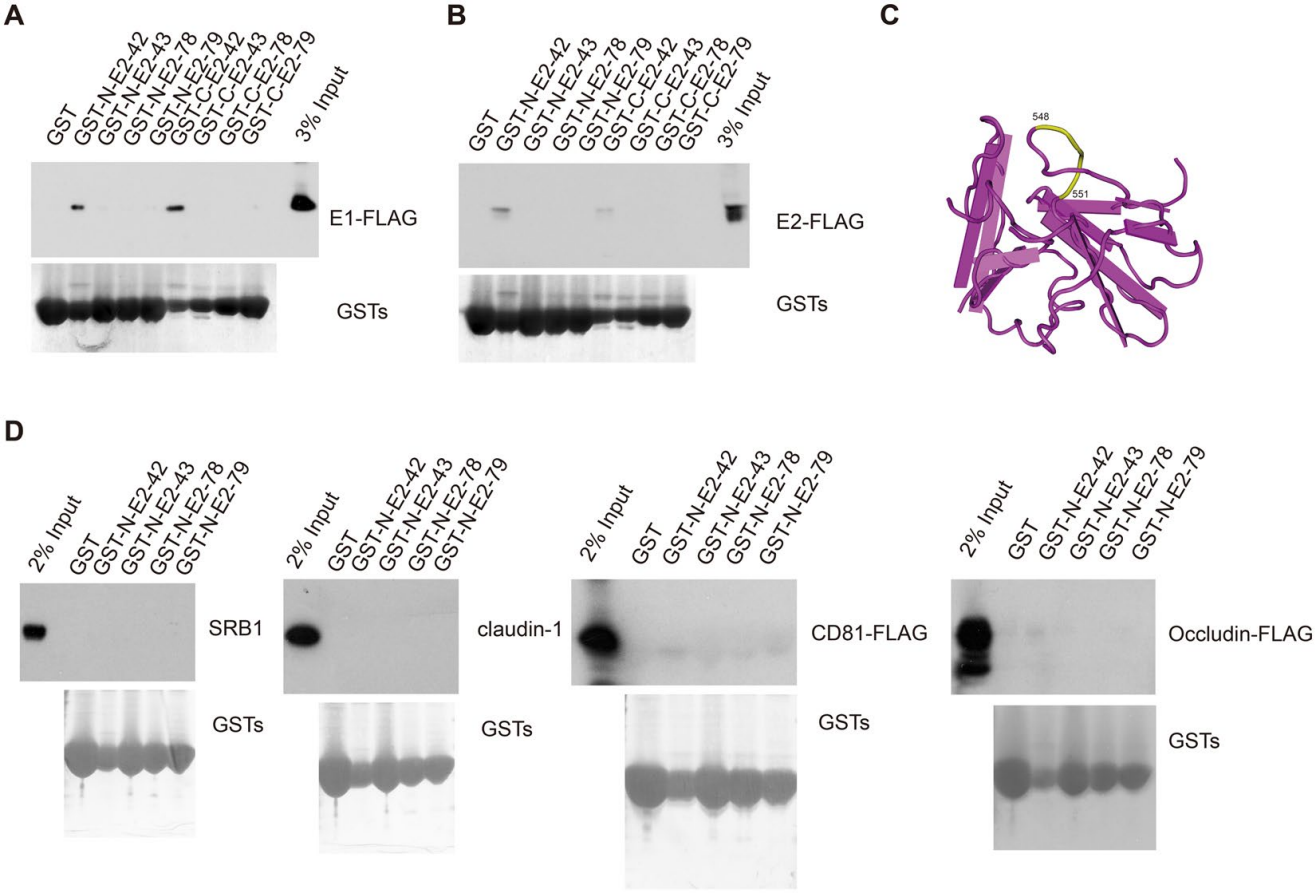
E2-42 interacts with HCV envelope proteins. (**A**) GST pulldown revealed that E2-42 associated with E1. Cell lysates from 293 T cells expressing JFH1 E1-FLAG were incubated with purified GST-N-E2-42, GST-N-E2-43, GST-N-E2-78, GST-N-E2-79, GST-C-E2-42, GST-C-E2-43, GST-C-E2-78, GST-N-E2-79 or GST, and the proteins were pulled down with glutathione-Sepharose beads. Bound proteins were detected by immunoblotting using anti-FLAG antibody. (**B**) GST pulldown identified an association between E2-42 and E2. Cell lysates from 293 T cells expressing JFH1 E2-FLAG were incubated with purified GST-N-E2-42, GST-N-E2-43, GST-N-E2-78, GST-N-E2-79, GST-C-E2-42, GST-C-E2-43, GST-C-E2-78, GST-N-E2-79 or GST, and the proteins were pulled down with glutathione-Sepharose beads. Bound proteins were detected by immunoblotting using anti-FLAG antibody. (**C**) Residues 548-551 of E2 is exposed in E2 structure. Ribbon representation of partial E2 model structure (PDB ID:4WHT) with residues 548–551 highlighted in yellow. (**D**) GST pulldown confirmed that E2-42 does not interact with HCV receptors. Cell lysates from Huh7.5.1 or 293 T cells expressing CD81-FLAG or occludin-FLAG were incubated with purified GST-N-E2-42, GST-N-E2-43, GST-N-E2-78, GST-N-E2-79 or GST, and the proteins were pulled down with glutathione-Sepharose beads.

On the surface of HCV particles, E1 forms a trimer in an E2-dependent manner, and both E1 and E2 are required for HCV entry^18^. Because E2-42, E2-43, E2-78 and E2-79 blocked HCV entry when the peptides were present during and after virus infection, we investigated whether these peptides interact with HCV envelope proteins using a GST pull-down assay. As shown in Fig. 6A and B, E1 and E2 associated with GST-E42 and E42-GST. Compared with E2-42 (residue 548–562), E2-43 (residue 552–566) is lack of sequence from 548 to 551, which is predicted to exposed to the surface and therefore a potential interaction site for E1 and E2 (Fig. 6C). However, the four peptides did not interact with HCV receptors (SRB1, claundin-1, CD81 and occludin) (Fig. 6D), consistent with the lack of antiviral activity of these four peptides when incubated with the cells before infection.

## Discussion

This study describes a novel strategy to screen HCV inhibitors. We synthesized 126 peptides covering the HCV genotype 2a strain JFH-1 envelope glycoprotein E1E2. Each peptide consisted of 15 amino acids (AA) composed of two adjacent overlapping 10-AA (for E1) or 11-AA (for E2) regions. By screening the inhibition of HCVcc infection, we identified four novel anti-HCV peptides located in E2 (E2-42, E2-43, E2-78, E2-79). However, the screening might miss some inhibitory peptides because those peptides are not totally overlapped. These novel peptides strongly inhibited HCV independent of genotype. Interestingly, E2-42 associated with E1 and E2. Our results indicated that these four peptides inhibit HCV entry at the post-attachment step and block cell-to-cell transmission. Peptide E2-42 binding to E1 or E2 may also inhibit the late steps of HCV life cycle, such as HCV package and release. These inhibitory peptides might be used to treat HCV in combination with other anti-HCV agents.

Viral entry is the first step of the HCV life cycle^14^. Our study demonstrated that the four peptides inhibit HCVcc entry at the post-attachment step in a pan-genotypic manner. E2-42, E2-43, E2-78, and E2-79 exhibited broad-spectrum inhibitory effects against different genotypes of HCVcc, including gene type 2a: Jc1p7NS2Gluc2a and J6CGluc2a; genotype 1a/2a: H77/JFHGluc2a; and genotype 1a/2a: HB/JFHGluc2a. The inhibition rate of these peptides for genotype 2a, genotype 1a/2a, genotype 1b/2a chimeric virus infection was 80%, 75% to 80%, and 60% to 70%, respectively. The inhibitory effects of peptides E2-42 and E2-43 were slightly greater than those of peptides E2-78 and E2-79.

In this study, we attempted to investigate the inhibitory mechanisms of E42/E42/E78/E79 on HCV. These peptides did not associate with HCV receptors, including CD81, SRB1, claudin 1, and occludin. Our data demonstrate that E42/E42/E78/E79 have no effects on HCV viral RNA replication. We speculate that E42 may block viral entry and spread via E1 and E2. Further studies are needed to understand the inhibitory mechanisms of E42/E42/E78/E79.

The design of synthetic peptides for inhibition of viral infection is a classic strategy. For example, Enfuvirtide, a 36-AA peptide derived from the HIV virus envelope glycoprotein gp41 transmembrane region inhibits HIV-1 entry and has been used for clinical treatment of HIV-1-infected patients^19^. Several groups have identified HCV entry inhibitory peptides derived from E1E2^15, 20^. Consistent with these findings, we observed that peptides from similar regions, such as E1-17, E1-18, E1-27, and E2-28, inhibit HCV entry, which suggests that our methods are feasible. During the preparation of our manuscript, a study reported that the region covering E2-78 and E2-79 plays a role in inhibiting HCV entry^21^. We narrowed down the inhibitory peptides in this area to 15 AA, shorter than the peptide in the previous study. Our screen identified E2-42 and E2-43 as two novel entry inhibitory peptides. We were able to identify E2-42 and E2-43 because we used 15-AA peptides, while the other group used longer peptides.

The four peptides exhibited significant combination inhibition of HCV infection in combination with IFN-α2b or anti-CD81 monoclonal antibody in a dose-dependent manner. In addition, the combination of inhibitory peptides and IFN-α2b or CD81 monoclonal antibody had a broad-spectrum inhibitory effect on HCV infection. In the future, we will optimize the structure and amino acid composition of the inhibitory peptides to improve their inhibition efficiency. We will also combine the peptides with DAAs to inhibit HCV infection and thus provide a reference for improving clinical HCV combination therapy strategies.

In conclusion, we identified and characterized four new E2-derived HCV inhibitors. These inhibitors blocked HCV entry and suppressed HCV spread in cell culture, suggesting that they are strong candidates for further antiviral drug development. Furthermore, we determined that one of these peptides (E2-42), interacts with E1/E2; this interaction might play a role in the inhibition of HCV infection, opening a window to explore potential mechanisms of HCV entry.

## Methods

### Cells and viruses

The cell line Huh7.5/CD81, a derivative of Huh7 highly permissive for HCV-RNA replication and infection, was cultured in Dulbecco’s modified Eagle’s medium (DMEM) (Invitrogen, Carlsbad, CA, USA) with 10% fetal bovine serum (FBS) (Invitrogen, Carlsbad, CA, USA), nonessential amino acids (Invitrogen, Carlsbad, CA, USA), 100 g/mL streptomycin (Invitrogen, Carlsbad, CA, USA), 100 IU/mL penicillin (Invitrogen, Carlsbad, CA, USA), and 2 mmol/L L-glutamine (Invitrogen, Carlsbad, CA, USA). The HCV JFH1 Luc-NS3-5B replicon-containing cell line was provided by Dr. Shan Cen (Institute of Medicinal Biotechnology, Chinese Academy of Medical Sciences and Peking Union Medical College, Beijing). pFLJc1p7NS2Gluc2a was a gift from Dr. Charles Rice (Rockefeller University, NYC, NY, USA). pFLHB/JFHGluc2a, pFLH77/JFHGluc2a and pFLJ6/JFHGluc2 were previously constructed in our lab^22^.

### Antibodies

The primary mouse antibodies included anti-HCV core (Thermo, Waltham, MA, USA), anti-HCV NS5A (ViroGen Co, Watertown, MA, USA), anti-SRB1 antibody (BD, Franklin Lakes, NJ, USA), and anti-FLAG antibody (Sigma-Aldrich, St. Louis, MO, USA). The primary rabbit antibody was anti-claudin-1 antibody (Abcam, Cambridge, MA, USA). Secondary antibodies included HRP-conjugated ECL goat anti-rabbit IgG, HRP-conjugated ECL goat anti-mouse IgG (Amersham Biosciences, Piscataway, NJ, USA), and goat anti-mouse IgG conjugated with AlexaFluor 594 (Invitrogen, Carlsbad, CA, USA).

### Synthesis of peptides

A total of 126 peptides derived from the HCV genotype 2a JFH1 strain (GenBank no. AB047639) protein E1E2 were synthesized, of which 37 peptides were from HCV E1 and 89 peptides were from E2. Each peptide contained 15 amino acids, with 10 (for E1) or 11 (E2) amino acids of overlap between adjacent peptides.

### Production of cell culture-grown HCV (HCVcc)

pFLJc1p7NS2Gluc2a, pFLHB/JFHGluc2a, pFLH77/JFHGluc2a and pFLJ6/JFHGluc2 were linearized, further purified with ethanol and sodium acetate, and used as templates for *in vitro* RNA synthesis using the RiboMAX™ Large Scale RNA Production System-T7. The synthesized HCV subgenomic RNA was treated with DNase I (Promega, Madison, WI, USA), followed by acid phenol extraction to remove any remaining template DNA. Then, 10 µg of RNA was electroporated into 2 × 10^5^ Huh7.5/CD81 cells (270 V, 970 μF), and 48 h later, the cell culture supernatants were filtered through 0.1-µm filters (Millipore, Billerica, MA, USA) and collected.

### HCV infection assay

Huh7.5/CD81 cells were seeded in 48-well plates at a density of 1 × 10^4^/well before infection. The next day, the cells were infected with MOI = 0.05 focus forming unit (FFU)/cell in triplicate. Luciferase activity or HCV core expression level was assayed 72 h later.

### Cytotoxicity assay

Cell viability was determined using the MTT Cell Viability Assay Kit from R&D Systems (Minneapolis, MN, USA) following the manufacturer’s instructions. Briefly, aliquots of 1.5 × 10^4^ Huh7.5/CD81 cells/well were cultured in 96-well plates with fresh medium or medium with increasing concentrations of peptides for 48 h. The absorbance at 490 nm was measured with a 96-well plate reader. Each experiment was repeated at least three times.

### Luciferase reporter assays

Huh7.5.1/CD81 cells were infected with HCVcc for 1 h, and Gaussia luciferase activity was assayed 72 h later (Promega, Madison, WI, USA).

### DNA constructs

The DNA sequences encoding four peptides from E2 (GST-E2-N-42, E2-N-43, E2-N-78, E2-N-79) fused to the carboxyl terminus of GST were inserted into the EcoRI and XhoI sites of the pGEX4T-1 vector. The PCR primers were as follows: 5′-AATTCCAGGGCTCATGGTTCGGCTGCACGTGGATGAACTCCACTGGTTTCTGAC-3′ (GST-E2-N-42 forward); 5′-TCGAGTCAGAAACCAGTGGAGTTCATCCACGTGCAGCCGAACCATGAGCCCTGG-3′ (GST-E2-N-42 reverse); 5′-AATTCTTCGGCTGCACGTGGATGAACTCCACTGGTTTCACCAAGACTTGTTGAC-3′(GST-E2-N-43 forward); 5′-TCGAGTCAA CAAGTCTTGGTGAAACCAGTGGAGTTCATCCACGTGCAGCCGAAG-3′(GST-E2-N-43 reverse); 5′-AATTCGGTCTTCTCCACCTTCACCAGAACATCGTGGACGTACAATACATGTGAC-3′ (GST-E2-N-78 forward); 5′-TCGAGTCACATGTATTGTACGTCCACGATGTTCTGGTGAAGGTGGAGAAGACCG-3′(GST-E2-N-78 reverse); 5′-AATTCCTTCACCAGAACATCGTGGACGTACAATACATGTATGGCCTCTCATGAC-3′(GST-E2-N-79 forward); 5′-TCGAGTCATGAGAGGCCATACATGTATTGTACGTCCACGATGTTCTGGTGAAGG-3′ (GST-E2-N-79 reverse). The DNA sequences encoding four peptides from E2 (E2-C-42, E2-C-43, E2-C-78, E2-C-79) fused to the amino terminus of GST were inserted into the NcoI and SacI sites of pETGEX-CT. The PCR primers were as follows: 5′-GATGGAGCAGGGCTCATGGTTCGGCTGCACGTGGATGAACTCCACTGGTTTCGAGCT-3′ (GST-E2-C-42 forward); 5′-CGAAACCAGTGGAGTTCATCCACGTGCAGCCGAACC ATGAGCCCTGCTC-3′(GST-E2-C-42 reverse); 5′-GATGGAGTTCGGCTGCACGTGGA TGAACTCCACTGGTTTCACCAAGACTTGTGAGCT-3′(GST-E2-C-43 forward); 5′-CACAAGTCTTGGTGAAACCAGTGGAGTTCATCCACGTGCAGCCGAACTC-3′(GST-E2-C-43 reverse); 5′-GATGGAGGGTCTTCTCCACCTTCACCAGAACATCGTGGACGTAC AATACATGGAGCT-3′(GST-E2-C-78 forward); 5′-CCATGTATTGTACGTCCACGATGT TCTGGTGAAGGTGGAGAAGACCCTC-3′(GST-E2-C-78 reverse); 5′-GATGGAGCTTC ACCAGAACATCGTGGACGTACAATACATGTATGGCCT CTCAGAGCT-3′(GST-E2-C-79 forward); 5′-CTGAGAGGCCATACATGTATTGTACG TCCACGATGTTCTGGTGAAGCTC-3′ (GST-E2-C-79 reverse). Constructs encoding CD81 were inserted into the EcoRI and XbaI sites of pCMV14-3XFLAG. The PCR primers were as follows: 5′-CCCAAGCTTATGTCCGGATCCTGGCTCCGCG-3′ (forward); 5′-GCTCTAGAGTAC ACGGAGCTGTTCCGG-3′ (forward). Constructs encoding occludin were inserted into the HindIII and XbaI sites of pCMV14-3XFLAG. The PCR primers were as follows: 5′-CCCAAGCTTATGTCATCCAGGCCTCTTG-3′ (forward) and 5′-GCTCTAGATGTTTTCTGTCTATCATAG-3′ (reverse).

### GST pulldown

Expression of GST protein or the GST-peptide fusion protein was induced by 0.5 mM IPTG in E. coli Rosetta (DE3) at 37 °C for 4 h. The cells were then lysed with lysis buffer (50 mM Tris pH 6.8, 1 mM EDTA, 100 mM NaCl), and GST or GST-fused proteins were purified using glutathione-Sepharose beads. For the pulldown assay, the cell lysates were incubated with GST or GST peptides. After incubation at 4 °C for 1.5 h, the glutathione beads were pelleted and washed three times with PBS buffer. The protein samples were then boiled in SDS-containing loading buffer for gel electrophoresis followed by western blotting.

### qRT-PCR analysis

HCV RNA was quantitated by qRT-PCR analysis. Briefly, RNA was isolated from the cell culture medium using TRIzol reagent (Invitrogen, Carlsbad, CA, USA) following the manufacturer’s protocol. qRT-PCR was performed on a C1000 thermal cycler (Bio-Rad, Hercules, CA, USA) with an artus HCV RG RT-PCR Kit (Qiagen, Germany). Each experiment was repeated at least three times.

### Western blot analysis

Huh7.5/CD81 cells were lysed in ice-cold RIPA buffer (50 mM Tris-HCl, pH 7.5, 150 mM NaCl, 1% Triton X-100, 0.1% SDS, and 0.5% sodium deoxycholate) supplemented with a protease inhibitor mixture (Sigma, St. Louis, MO, USA). The cell lysates were incubated on ice for 30 min, centrifuged, and resolved by 10% SDS-PAGE. The proteins were then transferred to a PVDF membrane (Pall, Port Washington, NY, USA), followed by blocking with 5% skim milk for 1 h and incubation with the selected primary antibody overnight at 4 °C. The following day, the membranes were incubated with the corresponding IRDye 800-labeled IgG secondary antibodies (Li-Cor Inc., Lincoln, NE, USA) and scanned using the Odyssey Infrared Imaging System (Li-Cor Inc., Lincoln, NE, USA).

### Immunofluorescence Microscopy

HCV cell-to-cell transmission assay was performed according the reference^23^ and measured by immunofluorescence microscopy. Cells infected with HCV were washed with PBS, fixed with 4% paraformaldehyde, and permeabilized with 0.2% Triton X-100. The fixed cells were blocked with 1% bovine serum albumin and 1% normal goat serum in PBS. HCV NS5A protein in cells was detected by incubation with mouse anti-NS5A monoclonal antibody (ViroGen, Watertown, MA, catalogue No. 256-A) and visualized with secondary goat anti-mouse IgG conjugated to AlexaFluor 594 dye (Invitrogen, Carlsbad, CA, USA). The cover slips were mounted on slides with 4′,6-diamidino-2-phenylindole (DAPI) (Invitrogen, Carlsbad, CA, USA), and the HCV proteins were visualized by fluorescence microscopy (Olympus, Japan).

### Statistical Analysis

The qRT-PCR, western blot and luciferase activity assays were performed in triplicate. The data were analyzed using a two-tailed paired Student’s t-test.
